# DEPRESSIVE COGNITIONS AND SYMPTOMS IN CAREGIVING GRANDMOTHERS: TESTING GROWTH CURVE MODEL STABILITY

**DOI:** 10.1093/geroni/igad104.2146

**Published:** 2023-12-21

**Authors:** Christopher Burant, Carol Musil, Jaclene Zauszniewski, Alexandra Jeanblanc

**Affiliations:** Case Western Reserve University, Cleveland, Ohio, United States; Case Western Reserve University, Cleveland, Ohio, United States; Case Western Reserve University, Cleveland, Ohio, United States; Case Western Reserve University, Cleveland, Ohio, United States

## Abstract

Grandmothers caring for grandchildren have elevated depressive symptoms levels compared to grandmothers who do not provide care. Depressive symptoms are state-like in nature and describe recent depressive symptoms. The Depressive Cognition Scale© captures changes in negative thinking patterns that often precede depressive symptoms. Depressive cognitions, according to Beck’s theory of depression, are the first depressive symptoms to appear and typically lead to more serious symptoms of depression. Previously diagnosed depression may also contribute to current levels of depressive cognitions and depressive symptoms. Data were collected on 342 participants in a longitudinal nationwide online research study of caregiving grandmothers. A latent growth curve model of how previously diagnosed depression and depressive cognitions impacted the trajectory of depressive symptoms at four time points (baseline, 2 weeks, 12 weeks, and 24 weeks) was tested. The model fit the data well (Chi Square=24.301.; df=12; p=.019; TLI=.977; CFI=.987; RMSEA=.055). This model was submitted to 6 separate multigroup nested model tests (levels of income, education, age, employment, number of children, and primary vs. multigeneration families) to provide evidence supporting generalizability of the model. All models fit the data well (TLI=.960-.991; CFI=.973-.992; RMSEA=.051-.025). Total invariance/stability, based on nonsignificant changes in chi square and CFI<.01, was supported in the multigroup analyses across all levels testing equality in measurement and regression weights and residual variances. This lack of change in the model across 6 different grandmother scenarios provides further evidence supporting the need for interventions to address diagnosed depression and depressive cognitions for reducing depressive symptoms in grandmother caregivers.

